# Clustering analysis of volatile organic compound biomarkers with tobacco exposure and the association with cardiovascular health outcomes in an observation study cohort

**DOI:** 10.18332/tid/200649

**Published:** 2025-05-16

**Authors:** Juan Zhao, Haoyun Hong, Joseph Zhai, Remy Poudel, Sanjay Srivastava, Andrew C. Stokes, Pawel K. Lorkiewicz, Tian Jiang, Rose Marie Robertson, Aruni Bhatnagar, Jennifer L. Hall, Naomi M. Hamburg, Rachel J. Keith

**Affiliations:** 1 American Heart Association, Dallas, United States; 2 American Heart Association Tobacco Regulation and Addiction Center, University of Louisville, Louisville, United States; 3 Department of Global Health, Boston University School of Public Health, Boston, United States; 4 Vascular Biology Section, Whitaker Cardiovascular Institute, Boston University School of Medicine, Boston, United States

**Keywords:** volatile organic compounds, e-cigarettes, combustible cigarettes, cardiovascular health, hazardous and potentially harmful chemicals

## Abstract

**INTRODUCTION:**

Volatile organic compounds (VOCs) are toxic compounds found in tobacco smoke. Despite research on cigarette generated single VOCs, scant evidence exists on the mixtures of VOCs associated with different tobacco products. We aimed to explore whether distinct VOC exposure profiles exist among users of combustible cigarettes, e-cigarettes, and non-users, and to assess their associations with cardiovascular (CV) health markers.

**METHODS:**

Participants who self-reported use of e-cigarettes, cigarettes, or no tobacco (n=348; mean age 26 ± 7 years) enrolled in The Cardiovascular Injury due to Tobacco Use (CITU) 2.0 study from July 2018 to July 2023 at two US sites (Boston, MA, and Louisville, KY). VOC metabolites were analyzed in urine one-hour post-use of a tobacco product via ultraperformance liquid chromatography. We applied unsupervised K-Means clustering on the creatinine-adjusted VOC metabolite data and explored the association between each cluster and blood pressure, adjusting for age, sex, and race.

**RESULTS:**

The clustering analysis identified two distinct clusters. Cluster 1 (302 individuals, 86.8%) was characterized by low VOC metabolite levels with individuals predominantly e-cigarette users (59.3%), non-users (29.1%), and a smaller proportion of cigarette smokers (11.6%). Cluster 2 (46 individuals, 13.2%) had higher levels of VOC metabolites including CYMA, HPMMA, MHBMA3, and 3HPMA, and included most of the individuals who used cigarettes (91.3%). After adjustment for age, sex, and race, Cluster 2 was associated with a higher heart rate (β=3.29; 95% CI: -0.26–6.84; p<0.05) compared to Cluster 1. No significant differences were observed for systolic (β= -0.66; 95% CI: -4.60–3.28) or diastolic blood pressure (β=0.34; 95% CI: -2.51–3.2) between clusters.

**CONCLUSIONS:**

These findings suggest that cigarette-induced VOC exposure may not impact cardiovascular function after acute exposure. Additionally, VOC exposure profiles vary across tobacco product types, suggesting that regulatory assessments of tobacco products could consider exposure patterns rather than product types. Clustering analyses may offer a powerful tool to assess the safety and risks of new and emerging tobacco products based on real-world exposure patterns.

## INTRODUCTION

Volatile organic compounds (VOCs) are ubiquitous hazardous and potentially hazardous chemicals that are emitted from various sources including industrial emissions and household products^1^, and are also found in high levels in tobacco products^2^. Tobacco-induced VOC exposure can be inhaled directly from active use or indirectly from secondhand exposure to tobacco products such as combusted tobacco^3^ and electronic cigarettes (e-cigarettes)^3,4^. These hazardous compounds have been associated with several health outcomes, including respiratory disease^5^, cancer^6^, and heart disease^7-9^. Understanding VOC exposure is critical to determining the health effects of tobacco products and potentially offering the FDA an innovative strategy to assess the risks of new tobacco products.

While existing research has largely focused on individual VOCs^6,10,11^, real-world exposure involves mixtures of multiple compounds, which may have a different effect as a mixture, for example synergistic or antagonistic effects on health. Analyzing VOCs individually overlooks these complex interactions, limiting our understanding of their cumulative impact. Identifying the exposure patterns from different tobacco products is crucial for policymakers to assess health risks and ensure appropriate product standards^12^. However, traditional regulatory approaches such as substantial equivalence focus on broad tobacco product classes with similar design features rather than the specific exposure patterns and risks posed by individual product use^13,14^.

With the growing understanding of the importance of mixture models there remains a gap in literature of mixture evaluation of the health effects caused by hazardous and potentially harmful compounds associated with tobacco products, such as VOCs^15-17^. Given the potential for additive health effects within mixtures, health assessments derived from single-source analyses may not capture real world relevance. Thus, it is important for policymakers to understand how different tobacco products and use patterns are associated with specific patterns of co-exposure.

This study aims to explore whether distinct VOC patterns are associated with the use of e-cigarettes and combustible cigarettes. Using data from the Cardiovascular Injury due to Tobacco Use 2.0 (CITU 2.0) study, a wide-ranging demographic cohort inclusive of individuals who currently use cigarettes, e-cigarettes, or have never used tobacco, we employed an unsupervised clustering method to identify VOC exposure profiles and assessed their association with cardiovascular disease (CVD) health markers.

## METHODS

### Study design

Participants were enrolled in the CITU 2.0 study from July 2018 to July 2023 at two US sites (Boston, MA, and Louisville KY). The participants were briefly self-reported healthy adults who currently used e-cigarettes (use for past 3 months and at least 3 times per week), cigarettes (>100 cigarettes and use for past 3 months and at least 3 times per week), cigarillos (use for past 3 months and at least 3 times per week) or non-users who had never used any tobacco products (<100 lifetime uses of any tobacco). Each institutional review board approved CITU 2.0 and all participants provided written consent (BU #H-32613 and UL #13.0590).

### Inclusion and exclusion criteria

At the time of conducting this analysis, 365 participants enrolled in the CITU 2.0 cohort. Exclusion criteria included missing demographic data needed for adjustment and use of cigarillos on the day of their visit or use of a product that may have contained cannabis instead of a nicotine vape liquid (Supplementary file Figure S1). We also excluded individuals without urinary measures of VOC measures at 1 hour post-exposure or without urinary creatinine. Participants who were part of the cigarillo groups were excluded because of the small sample size (n=7).

### Study protocol

Study visits were scheduled after an 8-h food fast and a 6-h tobacco fast. All study visits occurred before 11 a.m. to limit effects due to circadian changes. Each visit included a structured interview on demographics, socioeconomics, lifestyle, health, family history of heart disease, allergies, and tobacco use. Detailed self-reported tobacco use history was collected using a modified version of the National Health Interview survey on tobacco use^18^ and surveys harmonized with the PhenX toolkit to include detailed information on ENDS and non-traditional tobacco products. Fasting blood and urine samples were collected, as well as vascular health indices. All vascular function studies were completed after 10 min of supine positioning. A centralized laboratory at the University of Louisville processed and performed urinary and blood measurements. All surveys were collected and kept in Research Electronic Data Capture (REDCap), a secure web application for building and managing online surveys and databases^19,20^.

*Acute tobacco exposure session*

After completion of the baseline measures, participants were asked to complete a structured use protocol for smoking, vaping, or sham (those who do not use tobacco products) within our specially designed exposure rooms. Participants were asked to bring in their typical product for this session, with dual users bringing an e-cigarette. Those who would smoke were asked to use a completely combustible cigarette for ≤10 min. Those who would vape were asked to bring a new vape or filled device of their most common current product, flavor, and nicotine strength. They were then asked to complete one 3-s puff every 30 s for 10 min. If participants reported they were unable to handle the nicotine content from 2 puffs/min, they could reduce their puffs to 1/min. Those who did not use any tobacco products were asked to inhale on a straw for three seconds every 30 s for 10 min. Urine and vascular measures were collected as described one and two hours after the exposure session^21^.

### VOC measurements

Standard clean catch urine specimens were obtained from participants and stored at 4°C. The samples were transported for long-term storage and mass spectrometric analysis at the University of Louisville. A total of 23 urinary metabolites of tobacco-induced aldehydes and other VOCs were quantified with a modified version of the mass spectrometry method developed by the Centers for Disease Control and Prevention (CDC)^22^ and described in detail by Lorkiewicz et al.^23^. The analysis was performed on an ACQUITY UPLC core system and a Quattro Premier XE triple quadrupole mass spectrometer coupled with an electrospray source (Waters, Inc, MA). Urine (25 µL) was mixed with 15 mM ammonium acetate (975 µL) containing a mixture of internal standards and filtered through a 0.2 mm polytetrafluoroethylene membrane. Two microliters of the sample were applied on ACQUITY UPLC HSS T3 column (150 mm × 2.1 mm, 1.8 µm; Waters, Inc) maintained at 40°C and preequilibrated with ammonium acetate (15 mM, pH 6.8; solvent A) at a flow rate of 0.45 mL/min. The binary gradient started with 3% solvent A at 0 min and was linearly increased to 5% solvent B (acetonitrile) at 1.3 min, 10% B at 2.0 min, 30% B at 3.35 min, and 40% at 4.36 min. The gradient was then decreased to 15% B at 4.7 min, 10% B at 5.0 min, and 3% B at 5.36 min. The samples were analyzed, both in positive and negative ion modes. Three multiple reaction monitoring procedures were conducted for each analyte: one for quantification, one for confirmation, and one for stable isotope labeled analogous internal standard. At least 12 data points across the peaks were used for the quantitation of peak area. Analytes in urine samples were quantified using peak area ratio (analyte to internal standard) based on 10-point-standard curves that were run before and after the urine samples. TargetLynx quantification application manager software (Waters, Inc.) was used for peak integration, calibration, and quantification. The concentration ranges determined for this method are comparable to those reported in the CDC method for the VOC metabolites^22,23^. Similarly, the reproducibility of the method was satisfactory, with relative SDs below 8% for VOC metabolites and alkaloids (5.5% for cotinine). Additional validation shows comparability in terms of sensitivity, accuracy, and precision compared to the CDC.

The concentration values of analytes obtained from ultra-performance liquid chromatography-tandem mass spectrometer were normalized to the creatinine level, which was measured on a COBAS MIRA-plus analyzer (Roche, NJ) with Infinity Creatinine Reagent (Thermo Fisher Scientific, MA)^23^.

### Cluster analysis

We conducted a cluster analysis on VOC biomarkers from the CITU 2.0 data set, utilizing the KMeans algorithm to identify distinct biomarker profiles. VOC measurements that were obtained one hour after smoking were fed into the clustering method. To determine the optimal number of clusters that were derived, we used the Elbow method, and used principal component analysis (PCA) facilitated visualization of cluster differentiation. To ensure data quality, VOCs with incomplete records or invalid entries – such as 12DCVMA, TCVMA, AMCC, and CEMA – were excluded. Additionally, any VOCs with >85% of values below detection limits were removed from analysis. After this preprocessing, a total of 12 metabolites (i.e. AAMA, 3HPMA, 2HPMA, MA, DHBMA, MHBMA3, PGA, HPMMA, 2MHA, 34MHA, BMA, and CYMA) were finally included in the analysis (Supplementary file Table S1). For values below detection limits, imputation was performed using values close to zero. To adjust urine dilution effects, all VOC values were normalized by dividing them by the creatine levels measured at the corresponding urinary time point.

### Statistical analysis

Descriptive statistics were computed for each cluster. Univariate tests were performed to compare the difference between clusters with two-sample t-tests for continuous variables, and the Z-proportion test for categorical variables. To examine the association between the clusters and surrogate cardiovascular (CV) health outcomes (e.g. systolic and diastolic blood pressure, heart rate), we used generalized linear regression by adjusting age, gender, and race/ethnicity. We report the β co-efficient and 95% confidence interval (CI). The significance level was defined as a p<0.05.

## RESULTS

The study included 348 unique participants after applying the inclusion and exclusion criteria (Supplementary file Figure S1). The average age was 26.1± 7.1 years. Of the 348 participants, 77 were cigarette smokers, 183 were e-cigarette users, and 88 were non-users. Among the 348 participants, 54 were dual users, 53.2% were males, and 62.1% were nonHispanic White.

### Demographic and product use characteristics of clusters

The clustering process resulted in 2 distinct clusters based on VOC biomarker profiles (Supplementary file Figure S2). Table 1 and Supplementary file Figure S3 present the demographic and product use characteristics within each cluster. Most individuals in Cluster 1 (n=302) were e-cigarette users (59.3%), with a smaller number of cigarette smokers (11.6%) and a few non-users (29.1%). In contrast, Cluster 2, with 46 individuals, was composed mostly of cigarette users (91.3%), with few using e-cigarettes (8.7%), and none reported as non-users or dual users.

**Table 1:** Demographic characteristics for two clusters

*Characteristics*	*Cluster 1* *n (%)*	*Cluster 2* *n (%)*
**Sample size**, n	302	46
**Age** (years), median (IQR)	22 (7.0)	34 (9.75)*
**Sex**, Female	146 (48.2)	17 (37.0)
**Race**		
White	182 (60.3)	34 (73.9)
Black	17 (5.6)	8 (17.4)*
Asian	81 (26.8)	1 (2.2)*
Other	22 (7.3)	3 (6.5)
**Ethnicity**		
Hispanic, Latino or Spanish	38 (12.6)	0 (0.0)*
**Education level**		
Lower than high school	10 (3.4)	5 (11.1)*
High school	35 (11.9)	13 (28.9)*
Some college	113 (38.4)	11 (24.4)
College and higher	136 (46.3)	16 (35.6)
**Product type use**		
Cigarette	35 (11.6)	42 (91.3)*
E-cigarette	179 (59.3)	4 (8.7)*
Non-user	88 (29.1)	0 (0.0)*
Dual use	54 (17.9)	0 (0.0)*
**Outcomes**		
Systolic blood pressure (mmHg), median (IQR)	115.0 (17.0)	119.0 (15.5)*
Diastolic blood pressure (mmHG), median (IQR)	70.0 (12.0)	74.5 (13.8)*
Heart rate (bpm), median (IQR)	63.0 (13.0)	67 (13.0)*

Statistical test: Kruskal-Wallis for continuous variables; Z-proportion test for categorical variables. Significance level: p<0.05.

* Significant. IQR: interquartile range.

Cluster 1 and Cluster 2 also demonstrate notable differences in demographic characteristics. Participants in Cluster 1 are likely to be younger (median age 22 years) and comprise 48.2% biological females (Table 1). The majority self-reported their race as White (60.3%), followed by Asian (26.8%), and a small percentage as Black (5.6%). Most individuals in this cluster self-report as non-Hispanic. Education completed is varied, with 38.4% having some college education or higher (46.3%). In contrast, Cluster 2 tends to be older (median age 34 years) and consists of a smaller proportion of females (37.0%). The cluster has a larger percentage of individuals self-reported as White (73.9%) and Black (17.4%), but fewer as Asian (2.2%). No one from this cluster identifies as Hispanic, Latino, or Spanish. Cluster 2 also shows a lower proportion with some college education (24.4%) or higher degrees (35.6%).

In terms of health outcomes, Cluster 2 has higher median systolic (119.0 vs 115.0 mmHg), diastolic blood pressures (74.5 vs 70.0 mmHg), and heart rate (67 vs 63 bpm).

### VOCs profiles by cluster

**Figure 1:**
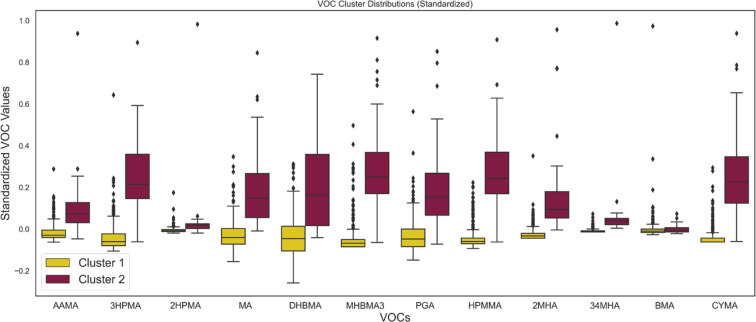
Box plots of standardized volatile organic compound (VOC) values across two identified clusters. The distribution of each VOC is summarized through its median, quartiles, and outliers. The plots highlight the variance within and between the clusters for each VOC, indicating the different exposure or metabolic profiles that characterize each cluster

Figure 1 shows the standardized VOC values among each cluster. Generally, Cluster 2 shows higher median values across most VOCs, particularly prominent in compounds such as DHBMA, MHB3A, and BMA. This indicates a higher concentration of VOCs presenting in Cluster 2, which could correlate with a higher level of tobacco exposure. When comparing VOC distributions to the usage patterns, the high representation of cigarette smokers in Cluster 2 aligns with the higher VOC levels in this cluster. Outliers are present in both clusters but are more prevalent in Cluster 2, indicating individual variations in VOC metabolite excretion.

**Figure 2:**
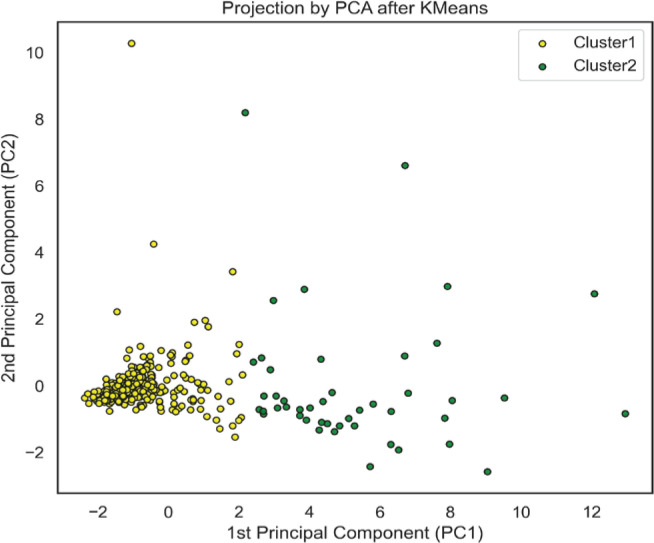
Scatter plot illustrating the distinction of two clusters in two dimensions of space through principal component analysis. The first principal component (PC1) captures the largest variance, the second principal component (PC2) accounts for the second-largest variance. Cluster 1 (yellow dots) is tightly grouped and its VOCs characteristics are more homogeneous than those of Cluster 2

We employed PCA along with a scatter plot to visualize the distinction between the two clusters within the first two principal component (PC) dimensions, as shown in Figure 2. The first two PCs explained 57.17% variance. Cluster 1 appears predominantly concentrated, suggesting that VOCs characteristic of this cluster is more homogeneous which differentiates them from Cluster 2. The PCA loading plot (Supplementary file Figure S4) reveals the VOCs contribute most to the differentiation. Specifically, compounds such as CYMA, HPMMA, MHBMA3, and 3HPMA exhibit high positive loadings on the first principal component (PC1), underscoring their role in differentiating Cluster 1. Conversely, BMA shows a high positive loading on PC2, suggesting its association with Cluster 1. This bimodal distribution underscores distinct biomarker profiles, potentially reflecting different tobacco exposure statuses within the population.

### Association between cluster and health outcomes

Table 2 contains the results of regression analysis that examined the association between health outcomes and two clusters, with Cluster 1 serving as the reference group. The unadjusted models indicated that Cluster 2 had significantly higher systolic blood pressure, diastolic blood pressure, and heart rate, with β coefficients of 4.21 (95% CI: 0.34–8.09), 3.70 (95% CI: 1.06–6.35) and 4.58 (95% CI: 1.36–7.79), respectively. After adjusting for age, gender, and race/ethnicity, the difference in heart rate was no longer statistically significant with a β of 3.29 (95% CI: -0.29–6.84).

**Table 2:** Association analysis between two clusters and the health outcomes using a generalized linear model

*Outcome*	*Cluster*	*Unadjusted* *β coefficients* *(95% CI)*	*Adjusted^a^* *β coefficients* *(95% CI)*
Systolic blood pressure	Cluster 1 ®		
	Cluster 2	4.21 (0.34–8.09)*	-0.66 (-4.60–3.28)
Diastolic blood pressure	Cluster 1 ®		
	Cluster 2	3.70 (1.06–6.35)*	0.34 (-2.51–3.20)
Heart rate	Cluster 1 ®		
	Cluster 2	4.58 (1.36–7.79)*	3.29 (-0.26–6.84)

a Adjusted for age, gender, and race/ethnicity. The significance level: p<0.05.

* Significant. ® Reference category.

## DISCUSSION

Our findings underscore the significance of considering VOC mixtures in regulatory assessments. This approach yields a mixture-based understanding of hazardous and potentially harmful compounds potential health risks and has substantial implications for public health policies aimed at tobacco regulation. Using clustering analysis of the mixtures of VOCs, we identified two distinct clusters based on statistical determination of the optimal number of groups. The two clusters revealed a demographic divergence in tobacco product usage, inherent VOC exposure, and health risk factors. Specifically, Cluster 1, was defined by lower median levels of VOCs and included younger individuals who either have never used tobacco or have used e-cigarettes. Cluster 2 was defined by higher median levels of VOCs and included older individuals who use traditional cigarettes. We further examined the association between clusters and CVD risk factors. We found that participants in Cluster 2 were likely to have a higher heart rate, even after adjusting for demographic variables.

Although the association of VOCs with traditional combustible tobacco is well-documented, the levels and combinations emitted by new products like e-cigarettes are less understood^4,3^. The generation of VOCs is frequently associated with combustion, as seen with combustible cigarettes. However, these compounds are found in numerous products unrelated to combustion. E-electronic cigarettes were introduced to the market as a reduced-harm product, mainly due to the elimination of combustion and the suggested health benefits of reducing hazardous and potentially hazardous compounds associated with combustion and tar. E-cigarettes eliminate combustion by utilizing a heating coil to vaporize nicotine-containing liquid, and though certain devices result in lower levels of VOCs^3,24^, others can produce VOC levels comparable to combustible cigarettes^25^. Our work suggests that the majority of our e-cigarette users had lower median VOC levels and tended to be closer aligned with a VOC profile of individuals with no tobacco exposure. For those e-cigarette users in Cluster 2, their use patterns and/or device may have exposed them to a greater level of VOCS thus grouping them into a cluster with a risk factor for CVD. Electronic cigarettes have changed rapidly since being introduced; thus, their exposure profile has changed over time and is likely to change in the future making these devices difficult to regulate as an overarching class. These findings suggest that individuals who use the same product class may have a different exposure profile and health risk, and thus the use of exposure patterns warrant consideration in future studies. From a regulatory standpoint, prioritizing the assessment of an individual’s exposure to hazardous and potentially harmful chemicals, rather than grouping a highly diverse range of devices, could be a crucial regulatory strategy in navigating the dynamic landscape of this market as well as evaluating new and emerging tobacco products.

Moreover, substantial equivalence which is used to bring new tobacco products to market addresses tobacco product classes with similar design features but does not consider specific mixtures of exposure. Additionally, health effects comparisons for the substantial equivalence products are based on single chemical exposure, losing the complexity and variable nature of hazardous and potentially harmful chemicals that would vary between and within product classes. Consequently, when tobacco product groups alone are used to define groupings for health evaluations, the categorization does not consider the varied individual exposure within a product family like e-cigarettes. As demonstrated in this study, individuals who used the same type of product were separated into different clusters, which had different health outcomes, by their VOC exposure pattern. Given that many tobacco regulatory decisions are based on product classes, which results in mixed exposure to harmful constituents that differentially impact health, it is important to develop and explore more robust models of identifying health risks.

Mixed-exposure research can address the additive effects of chemicals, which could be synergistic or antagonistic, and is imperative to understand in health models. Users of tobacco and e-cigarettes are always exposed to a mixture of hazardous and potentially harmful chemicals, but these types of exposures are exceedingly difficult to study from a statistical perspective^26^.

Research suggests that when modeling mixtures of chemicals, additive and independent compound analyses are a popular methodology, but do not adequately reflect the models of exposure. Consequently, it cannot account for the antagonistic effects of certain chemicals, such as the antagonistic relationship between CO_2_ and nicotine, seen with smoking. These models tend to underestimate the enhanced effects of mixtures on physiological responses^27^. Our study used cluster analysis to explore mixtures of VOCs and health outcomes. Our cluster analysis generally separated the cohort based on several key factors including age and product of use. Other statistical approaches to modeling mixtures of exposure are available but have not been rigorously applied to tobacco science, leaving a gap in the literature for appropriate mixed-model methodology.

Our findings underscore the critical role of socioeconomic and demographic factors in shaping exposure to tobacco-related VOCs^28-30^. For example, younger populations, favoring e-cigarettes over traditional tobacco products, may show distinct VOC exposure profiles compared to older populations, as indicated by age-related usage patterns. Cluster 1 (e-cigarettes and non-users) also shows a more diverse racial composition, with the majority identifying as White, followed by a notable proportion of Asian individuals, and a smaller percentage as Black participants. Age and race are crucial considerations in tobacco regulatory science due to their demonstrated impact on nicotine metabolism^31^. Investigating demographic characteristics are also associated with alterations in metabolism of other harmful and potentially harmful constituents (HPHCs) such as VOCs is essential for understanding health effects in a diverse population. We also showed that Cluster 2 had a higher percentage of participants with high school or lower level of education. Lower education level is associated with reduced risk perceptions regarding tobacco use. This may result in reduced interactions with public health messaging and limited awareness of the increased exposure to hazardous and potentially harmful chemicals that may occur with different forms of tobacco products.

### Strengths and limitations

This study possesses several strengths. The utilization of cluster analysis in this study enables the characterization of mixtures of toxic VOCs, rather than solely focusing on the products themselves. Our ability to group HPHCs into a mixture and assess the health effects is highly relevant to real-world exposures and could be an important tool to consider for future regulatory science. The clustering approach we used here offers a means to enhance regulatory knowledge regarding health effects based on mixed-exposure models. These models are likely to vary across different tobacco product classes and user profiles, as opposed to single compounds. Thus, this approach allows a more comprehensive evaluation of the concept of substantial equivalence for not only current products, but also future tobacco products trying to enter the market.

Our study design asked participants to use their preferred products, allowing evaluation of the most widely used products on the market. Study designs that provide a product are often limited because they are designed with products that are dated and may have lost popularity among users. This dynamic approach provided high-quality relevant data to the tobacco science field. Highly relevant exposures paired with an immediate assessment of CVD risk factors, such as heart rate and blood pressure, are highly generalizable. The health effects examined in our study are relevant to chronic disease and are reversible, allowing these biomarkers to reflect changes with exposure to new and emerging tobacco products, as well as transitions between them. These metrics provide a snapshot of potential long-term risks without the need for the lengthy follow-up required in prospective studies. Furthermore, given the potential for a prolonged latency period – often spanning ten years or more – between increased cardiovascular risk factors and an actual cardiac event, assessing these health endpoints enables the early detection of signs of cardiotoxicity, supporting timely decision-making regarding the health effects of new products.

While the cluster analysis offers an understanding of the potential risks for specific VOC mixtures, not just a product, there are limitations. The mixture of e-cigarette users and non-users into a single cluster could potentially mask the subtleties in health changes between these two product use groups. The rapidly changing landscape of e-cigarette technology and formulations also suggests that these findings may be time-sensitive and subject to further evolution, though we did capture the exposure profiles of the most widely used products at the time of the study. Finally, self-reported data on tobacco product use could be subject to bias. While our study did not identify significant associations between VOC exposure clusters and cardiovascular risk factors, we saw trends that warrant further investigation. Longitudinal studies would be required to investigate the temporal relationships between VOC exposure and health outcomes, allowing for causal inference.

## CONCLUSIONS

Our findings suggest that lower levels of mixtures of VOCs included both users of e-cigarettes and those who used no tobacco products. This indicates that e-cigarettes might be associated with a different VOC exposure profile compared to traditional cigarettes, but the inclusion of some e-cigarette users in Cluster 2 suggests a potential influence of individual use patterns on VOC levels, which needs further investigation. Future studies should consider longitudinal designs to track changes in VOC exposure over time, including more granular data collection methods to capture the frequency and intensity of tobacco product use. Such detailed analyses could provide clearer guidance for public health policy and regulatory measures aimed at reducing the harm from tobacco products.

## Supplementary Material



## Data Availability

The data supporting this research are available from the authors on reasonable request.
