# Supplementary material for: Clustering analysis of volatile organic compound biomarkers with tobacco exposure and the association with cardiovascular health outcomes in an observation study cohort
Source: Tob Induc Dis. 2025 May 16;23:10.18332/tid/200649. doi: 10.18332/tid/200649 (PMC12083078; doi:10.18332/tid/200649)
Supplement: Supplementary file 1 [file TID-23-64-s1.pdf]

Supplementary material for

Clustering Analysis of Volatile Organic Compound Biomarkers: to Identify Tobacco Exposure and Association with Cardiovascular Health Outcomes using an Observation Study Cohort

Juan Zhao, PhD[1], Haoyun Hong, BA[1], Joseph Zhai[1], Remy Poudel, MPH[1], Sanjay Srivastava, PhD[2], Andrew C. Stokes, PhD[3], Pawel Konrad Lorkiewicz, PhD[2], Tian Jiang, MS[1], Rose Marie Robertson, MD[1], Aruni Bhatnagar, PhD[2], Jennifer L. Hall, PhD[1] , Naomi Hamburg, MD[4], Rachel J. Keith, PhD[2]

1. American Heart Association, Dallas, TX, USA

2. American Heart Association Tobacco, Regulation and Addiction Center, University of Louisville School of Medicine, Louisville, KY

3. Department of Global Health, Boston University School of Public Health, Boston, MA

4. American Heart Association Tobacco, Regulation and Addiction Center, Vascular Biology Section, Whitaker Cardiovascular Institute, Boston University School of Medicine, Boston, MA.

CORRESPONDING AUTHOR:

Rachel J. Keith, PhD

American Heart Association Tobacco, Regulation and Addiction Center, University of Louisville School of Medicine, Louisville, KY

Rachel.keith@louisville.edu

Table S1.

**Abbreviation**	**Compound (Unit)**
AAMA	N-Acetyl-S-(2-carbamoylethyl)-l-cysteine (ng/ml)
**3HPMA**	N-Acetyl-S-(3-hydroxypropyl)-l-cysteine (ng/ml)
**2HPMA**	N-Acetyl-S-(2-hydroxypropyl)-l-cysteine (ng/ml)
**MA**	Maleic acid or Malonic acid (ng/ml)
**DHBMA**	N-Acetyl-S-(3,4-dihydroxybutyl)-l-cysteine (ng/ml)
**MHBMA3**	N-Acetyl-S-(4-hydroxy-2-buten-1-yl)-l-cysteine (ng/ml)
**PGA**	Phenylglyoxylic acid (ng/ml)
**HPMMA**	N-Acetyl-S-(3-hydroxypropyl-1-methyl)-l-cysteine (ng/ml)
**2MHA**	2-Methylhippuric acid (ng/ml)
**34MHA**	3-Methylhippuric acid+4-Methylhippuric acid (ng/ml)
**BMA**	N-Acetyl-S-(benzyl)-l-cysteine (ng/ml)
**CYMA**	N-Acetyl-S-(2-cyanoethyl)-l-cysteine (ng/ml)

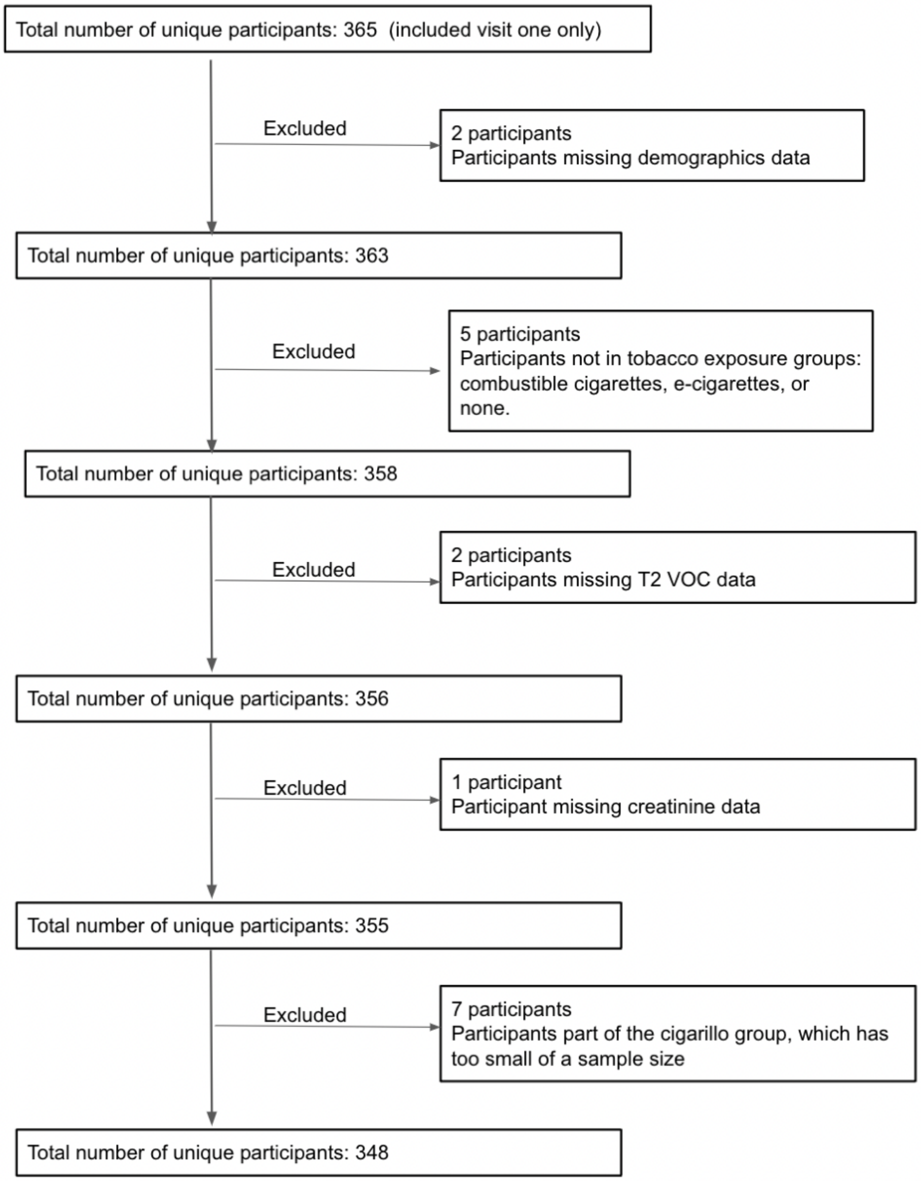

Figure S1. The flow diagram of study selection based on inclusion and exclusion criteria.

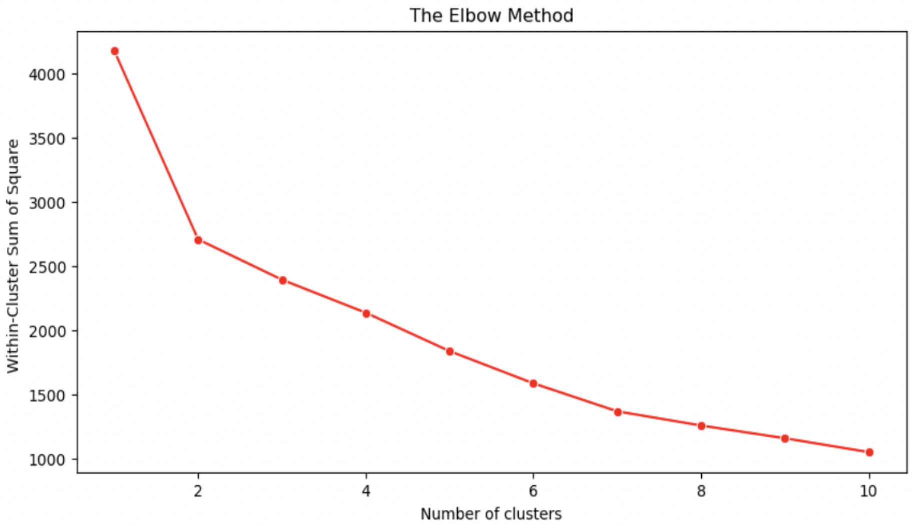

Figure S2. Elbow method for determining the number of clusters. This line chart illustrates the application of the Elbow Method to determine the optimal number of clusters for KMeans clustering. The y-axis represents the within-cluster sum of squares (WCSS), which measures the variance within each cluster, while the x-axis shows the number of clusters. The plot indicates a sharp decline in WCSS as the number of clusters increases from 1 to 2, with the rate of decrease diminishing after 2 clusters, suggesting that 2 clusters would be an optimal number.

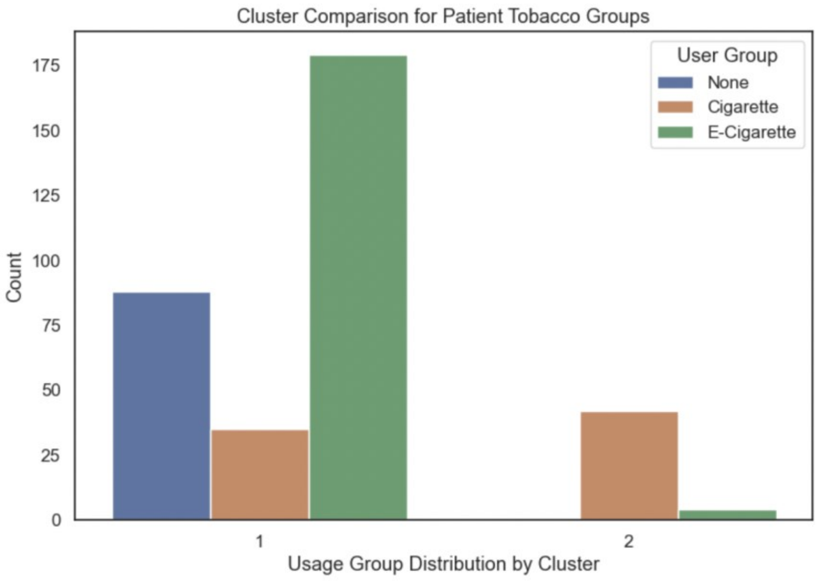

Figure S3. The distribution of patient tobacco usage among clusters. This bar chart illustrates the distribution of patient tobacco usage across two distinct clusters. Cluster 1 shows a higher prevalence of e-cigarette users (Green), with a small proportion of patients who do not use tobacco (Blue), and few cigarette smokers (Orange). In contrast, Cluster 2 predominantly consists of cigarette users (Orange), with very few patients using e-cigarettes or non-users. The data suggests a potential pattern in tobacco usage behavior among the different clusters.

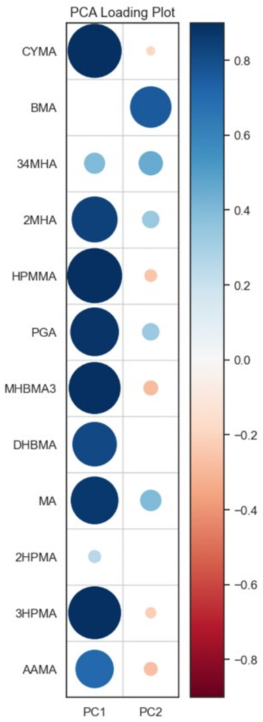

Figure S4. PCA loading plot. The PCA loading plot reveals the VOCs contributing most to the differentiation. Specifically, compounds such as CYMA, HPMMA, MHBMA3, and 3HPMA exhibit high positive loadings on the first principal component (PC1), underscoring their role in differentiating Cluster 1. Conversely, BMA shows a high positive loading on PC1, suggesting its association with Cluster 1. This bimodal distribution underscores distinct biomarker profiles, potentially reflecting different tobacco exposure statuses within the population.

© 2025 Zhao J. et al.
